# Predicting labor onset relative to the estimated date of delivery using smart ring physiological data

**DOI:** 10.1038/s41746-023-00902-y

**Published:** 2023-08-19

**Authors:** Elise N. Erickson, Neta Gotlieb, Leonardo M. Pereira, Leslie Myatt, Clara Mosquera-Lopez, Peter G. Jacobs

**Affiliations:** 1 College of Nursing / College of Pharmacy, The University of Arizona, Tucson, AZ USA; 2 Midwifery Division, School of Nursing, Oregon Health & Science University, Portland, OR USA; 3 Ouraring Inc, San Francisco, CA USA; 4 Department of Obstetrics & Gynecology, School of Medicine, Oregon Health & Science University, Portland, OR USA; 5 Artificial Intelligence for Medical Systems (AIMS) Lab, Department of Biomedical Engineering, Oregon Health & Science University, Portland, OR USA

**Keywords:** Predictive markers, Reproductive signs and symptoms

## Abstract

The transition from pregnancy into parturition is physiologically directed by maternal, fetal and placental tissues. We hypothesize that these processes may be reflected in maternal physiological metrics. We enrolled pregnant participants in the third-trimester (*n* = 118) to study continuously worn smart ring devices monitoring heart rate, heart rate variability, skin temperature, sleep and physical activity from negative temperature coefficient, 3-D accelerometer and infrared photoplethysmography sensors. Weekly surveys assessed labor symptoms, pain, fatigue and mood. We estimated the association between each metric, gestational age, and the likelihood of a participant’s labor beginning prior to (versus after) the clinical estimated delivery date (EDD) of 40.0 weeks with mixed effects regression. A boosted random forest was trained on the physiological metrics to predict pregnancies that naturally passed the EDD versus undergoing onset of labor prior to the EDD. Here we report that many raw sleep, activity, pain, fatigue and labor symptom metrics are correlated with gestational age. As gestational age advances, pregnant individuals have lower resting heart rate 0.357 beats/minute/week, 0.84 higher heart rate variability (milliseconds) and shorter durations of physical activity and sleep. Further, random forest predictions determine pregnancies that would pass the EDD with accuracy of 0.71 (area under the receiver operating curve). Self-reported symptoms of labor correlate with increased gestational age and not with the timing of labor (relative to EDD) or onset of spontaneous labor. The use of maternal smart ring-derived physiological data in the third-trimester may improve prediction of the natural duration of pregnancy relative to the EDD.

## Introduction

Only 5% of the nearly 4 million births occurring in the United States each year occur on the Estimated Date of Delivery (EDD), defined as 40 completed weeks from the first day of the last menstrual period^1,2^. Term pregnancy, where term labor is expected to begin, spans from 37–42 weeks of gestation and is typically associated with better outcomes than labors starting prior to the 37th week. The latter stages of pregnancy can be complicated by preeclampsia, problems with inadequate or excessive fetal growth (macrosomia) as well as concerns for fetal demise^3,4^. Obstetric care providers and families in their care, therefore, must balance these known risks of ongoing pregnancy with uncertainty and anxiety of when and where labor will start^5–8^. This may be particularly worrisome for those living in rural areas or in expanding ‘maternal care deserts’^9^ where residents may need to drive hours to appropriate hospital facilities^10^.

Symptoms signaling labor onset are difficult to precisely define as the experience of labor is highly variable between people^11^. As clinicians have no reliable way to predict when labor will begin, labor induction is routinely used in situations where uncertainty with waiting for labor could be risky, for example, living far from a hospital, developing an obstetric problem, or in cases where there is higher potential for a problem like hypertension or fetal demise occurring. For all of these reasons, nearly one-third (31.4%)^12^ of labors are induced; a rate that has more than tripled since 1990 (9.6%)^13^. However, it has been hypothesized that peripheral measures of autonomic activity, reflecting the neuroendocrine state with shifts in inflammation^14^ and steroidogenesis^15^ in the time preceding labor, may therefore be useful in understanding the shift between pregnancy to labor^16^. Few studies have examined these kinds of metrics in humans, though animal literature notes that changes in parasympathetic activity^17^ and body temperature fluctuate in advance of giving birth to offspring^18–23^.

In this study, we report on data derived from a multi-modal smart ring, worn by pregnant participants on a finger. We describe the change in physiological metrics across advancing gestation, and present results from a boosted random forest that was trained to predict whether pregnancies will extend beyond the EDD compared to those with a naturally shorter length of gestation. We present the derived physiological metrics that were most useful in improving prediction accuracy for the random forest. We also describe labor symptom frequencies across gestation and relative to timing of onset of labor.

## Results

### Participant characteristics

**Fig. 1:**
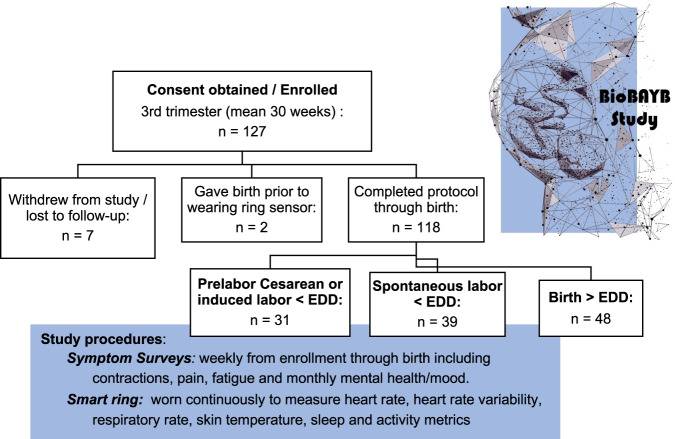
Flow diagram of participants and study procedures for BioBAYB study. Of the 127 participants gave consent and enrolled into the study, 118 completed data collection with prelabor/birth data from the wearable smart ring. Comparison groups for analysis included those who gave birth prior to the Estimated Date of Delivery (EDD) through planned prelabor Cesarean or induced labor, those who experienced labor starting before the EDD and those whose pregnancies lasted beyond the clinical EDD. Study procedures listed in blue box, sent via REDCap questionnaires, smart ring metrics gathered.

From July 2021 through April 2022, 127 pregnant participants living in the United States enrolled in the Biological Rhythms Before and After Your Birth (BioBAYB) Study. Six participants withdrew after consenting and one was lost to follow-up. Two participants enrolled and then underwent onset of labor prior to obtaining or wearing the device. For the remaining 118, the mean (standard deviation) maternal age on enrollment was 32.6 (4.1) years and the gestational age at enrollment was 30.3 (2.9) weeks (Fig. 1). Nulliparous individuals (never given birth previously) comprised 57.1% (*n* = 68) of the sample. The mean pre-pregnancy body mass index was 24.0 kg/m^2^ (SD 4.1). The majority had employer-based health insurance (*n* = 102) and had at least an undergraduate degree of education (*n* = 112, 94.6%). All participants reported being partnered or married. Six participants (4.8%) had a history of prior preterm birth and were enrolled slightly earlier in pregnancy, at 27.0 weeks. Timing of enrollment for the majority of the sample was intended to limit to those without gestational diabetes, which was tested around 26–28 weeks of gestation. Other baseline health and demographic characteristics are reported in Table 1.

**Table 1:** Demographic, pregnancy and birth characteristics of the BioBAYB study participants.

	*n* = 31 Induced Labor or Planned Cesarean Before EDD *n* (%)	*n* = 39 Spontaneous Labor Before EDD *n* (%)	*n* = 48 Birth after EDD *n* (%)	Test Two-tailed *t*/χ^2^	*p*
Age, years, mean (SD)	32.7 (4.3)	32.5 (4.3)	32.6 (3.8)	0.07	0.94
Self-reported race/ethnicity/ancestry (may have selected more than one)					
White/European	29 (90.6)	32 (82.1)	46 (95.8)	4.41	0.04
Asian or Pacific Islands	2 (6.3)	3 (7.7)	6 (8.4)		
Latin American/ Hispanic	2 (6.3)	7 (17.9)	1 (2.1)		
Black/African	0 (0)	2 (5.1)	0 (0)		
Other/Chose not to answer	1 (3.1)	2 (5.1)	1 (2.1)		
Gestational age at enrollment, weeks	29.4 (3.1)	29.9 (2.8)	31.2 (2.6)	2.14	0.04
Nulliparous	26 (81.3)	18 (46.2)	24 (50.0)	0.13	0.72
Pre-pregnancy body mass index kg/m^2^	24.6 (4.3)	23.9 (4.1)	23.7 (4.1)	−0.23	0.82
Medication/substances in pregnancy					
Daily aspirin	5 (15.6)	4 (10.3)	7 (14.6)	0.36	0.55
Anti-depressant (SSRI)	4 (12.5)	4 (10.3)	3 (6.3)	0.47	0.49
Iron supplement	8 (25.0)	10 (25.6)	21 (43.8)	3.08	0.08
Prenatal multivitamin	30 (93.8)	39 (100.0)	47 (97.9)	0.82	0.37
Fish oil supplement	13 (40.6)	22 (56.4)	18 (37.5)	3.10	0.08
Acetaminophen >3 times	9 (28.1)	5 (12.8)	9 (18.8)	0.56	0.45
Thyroid medication	0 (0)	4 (10.3)	5 (10.4)	0.0006	0.98
Sleep aids	5(15.6)	2 (5.1)	4 (8.3)	0.34	0.56
Edinburgh Postnatal Depression (EPDS)	6.6 (3.2)	5.6 (2.5)	5.3 (2.2)	−0.65	0.51
Generalized Anxiety Disorder (GAD 7)	3.5 (3.30	3.2 (2.5)	2.4 (1.9)	−1.58	0.12
Febrile illness, influenza, SARS-CoV-2	4 (12.5)	7 (17.5)	14 (29.2)	2.31	0.13
Pregnancy conditions after enrollment					
Hypertension or preeclampsia	4 (12.9)	2 (5.0)	2 (4.2)	0.05	0.83
Oligohydramnios/polyhydramnios	2 (6.3)	0 (0)	0 (0)		
Fetal growth restriction	4 (12.5)	1 (2.5)	1 (2.1)	0.02	0.88
Weight gained during pregnancy (lbs.)	36.9 (11.9)	34.1 (12.9)	34.1 (10.8)		
Labor onset					
Spontaneous labor	0 (0)	39 (100)	31 (64.6)	17.17	<0.001
Labor induction	27 (87.5)	0 (0)	17 (35.4)		
Pre-labor rupture of membranes	5		2		
Medical/obstetric condition	20		15		
Elective	3		1		
No Labor: Cesarean before labor	4 (12.5)	0 (0)	0 (0)		
Location of birth					
Hospital	32 (100)	30 (76.9)	40 (83.3)	1.27	0.53
Home (planned/unplanned)		5 (13.8)*	6 (12.5)		
Freestanding birth center		4 (10.3)	2 (4.2)		
Mode of birth					
Vaginal birth	21 (67.4)	32 (82.1)	36 (75.0)	1.41	0.49
Assisted vaginal birth	1 (3.1)	1 (2.6)	4 (8.3)		
Cesarean	9 (29.1)	6 (15.4)	8 (16.7)		
Duration of labor, hours (mean, SD)	14.1 (12.1)	19.0 (17.7)	16.3 (12.0)	−0.68	0.49
Infant weight, lbs. (mean, SD)	7.0 (0.9)	7.5 (0.9)	8.1 (1.0)	2.75	0.007
Gestational age birth, weeks (mean, SD)	38.7 (1.0)	38.9 (1.1)	40.8 (0.55)	10.44	<0.001
Labor and birth complications					
Umbilical cord prolapse	1 (2.5)	1 (2.5)	0 (0)		
Fever/infection	4 (12.5)	2 (5)	1 (2.1)	0.59	0.44
Received antibiotics during labor	6 (19.3)	3 (7.7)	11 (22.9)	3.69	0.06
Postpartum hemorrhage	2 (6.3)	2 (5.1)	4 (8.3)	0.34	0.56
Neonatal intensive care unit	4 (12.5)	6 (15.4)	3 (6.3)	1.94	0.16

Data grouped by timing of delivery relative to the Estimated Delivery Date (EDD) of 40.0 weeks of gestation. Bivariate tests, *p*-values compare spontaneous labor (*n* = 39) prior to the EDD to births occurring after the EDD (*n* = 48). No differences were observed in insurance provider, fetal sex or educational attainment.

*SSRI* selective serotonin reuptake inhibitor.

*n = 1 home birth was unplanned.

**Fig. 2:**
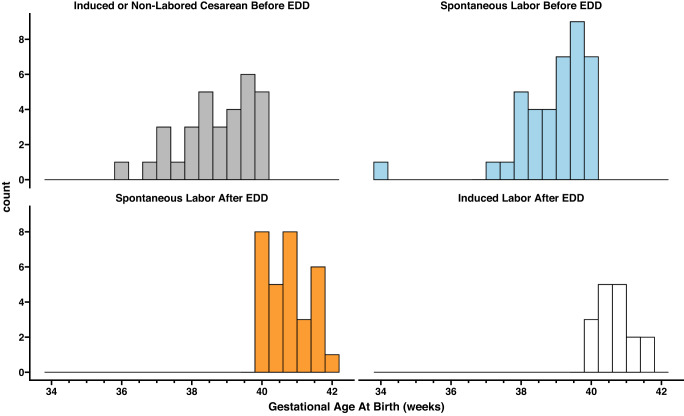
Distribution of the gestational age at delivery relative to timing of labor onset among 118 pregnancies enrolled in BioBAYB. Grey bars represent those with labor induction or pre-labor cesarean birth prior to the EDD. Blue bars denote spontaneous labor onset prior to the EDD. Orange bars denote the pregnancy passing the EDD with eventual spontaneous labor (*n* = 31) and white bars were labor inductions after the EDD (*n* = 17). No pre-labor cesarean births occurred among the births after the EDD.

Among participants who completed the after-birth survey, 8 (6.7%) reported developing gestational hypertension or preeclampsia after enrolling in the study. Spontaneous onset of labor occurred for 70 (58.8%) participants at a mean (standard deviation) gestational age of 39.8 (1.3) weeks and a range of 34.1–41.9 weeks. Only one preterm birth was reported (a twin gestation) when labor began spontaneously at 34 weeks of gestation. Among individuals who underwent labor induction, seven were performed for pre-labor spontaneous rupture of membranes (SROM) without a clear indication of labor symptoms or cervical dilation at the time of induction; labor induction occurred prior to the EDD for five of these seven participants. For the additional 41 labor inductions, nine were attributed to passing the due date (range of 40.1–41.1 weeks). Only four (3.3%) pregnancies ended with a Cesarean prior to labor onset due to obstetric indications (e.g., breech presentation). Figure 2 denotes the distribution and characterization of the gestational age at delivery and timing as well as the mode of labor onset relative to the EDD.

### Physiological metrics

Thirty metrics were available for analysis from the smart ring. For activity metrics, these data were available as averages across a 24-hour period. Sleep metrics represent an average during the sleep period. Waveform data was not available for analysis; thus, the metrics are based on the manufacturer’s algorithms for transforming or interpreting the data (e.g., sleep state or heart rate variability). The mixed effects regression revealed that a number of the daily physiological metrics acquired from the ring were significantly related with advancing days of gestation. Results from the regression analysis for each physical activity and sleep metric are shown (Table 2).

**Table 2:** Correlation of daily activity-based or sleep-based physiological metrics and gestational age in days.

Metric	Intercept (mean)	β(mean)	95% CI	*p*-value
Resting heart rate	72.33	−0.051	−0.056 to −0.046	*1.00E-84*
RMSSD	17.35	0.12	0.10–0.13	*2.46E-79*
Heart rate average	76.24	−0.038	−0.043 to −0.033	*1.72E-50*
Sleep efficiency	106.83	−0.072	−0.082 to −0.062	*2.11E-44*
Total sleep	36653	−21.77	−27.74 to −15.79	*1.04E-12*
REM	11067	−10.87	−13.90 to −7.83	*2.49E-12*
Inactive	269.78	0.70	0.49–0.90	*2.38E-11*
Respiratory rate	18.34	−0.0023	−0.0030 to −0.0015	*3.81E-09*
MET medium	172.77	−0.28	−0.39 to −0.17	*1.50E-06*
Calories active	363.33	−0.53	−0.75 to −0.31	*2.84E-06*
Daily movement	9232	−9.59	−13.81 to −5.36	*8.83E-06*
Bed start	−3515	16.67	9.17–24.17	*1.34E-05*
Medium activity	46.00	−0.07	−0.11 to −0.04	*1.35E-05*
Bed end	32603	14.49	7.78–21.20	*2.36E-05*
Deep sleep	2209	5.19	2.74–7.63	*3.27E-05*
MET inactive	8.77	−0.012	−0.017 to −0.006	*5.08E-05*
Temp deviation	−0.19	0.0005	0.0003–0.0007	*5.23E-05*
Caloric expenditure	1534.5	−0.57	−0.86 to −0.27	*0.000151*
Low activity	278.96	−0.20	−0.34 to −0.07	0.003146
MET	1.43	−0.00024	−0.00041 to −0.00007	0.006786
MET high	40.22	−0.08	−0.14 to −0.02	0.007032
Steps	8456.8	−5.47	−9.62 to −1.32	0.009766
Rest time	636.7	−0.21	−0.38 to −0.05	0.012659
High activity	4.70	−0.01	−0.02 to −0.0017	0.017516
MET low	196.97	−0.09	−0.19–0.01	0.067587
Non-wear time	230.74	−0.22	−0.54–0.11	0.196156
Temperature trend	−0.03	−0.00009	−0.00025–0.00008	0.311622
Restless sleep	24.34	0.0045	−0.0043–0.0132	0.320527
Sleep midpoint	18407.8	−1.18	−5.09–2.74	0.555642
Sleep onset	1096.21	−0.14	−1.13–0.86	0.790827

Metrics sorted by *p*-value from smallest to largest using mixed effects regression with the individual participant as the random effect, adjusted for pre-pregnancy body mass index, age, parity and sex of fetus. Significance was determined to be *p* < 0.002 (shown as italic).

*RMSSD* root mean square of successive differences, *REM* rapid eye movement, *MET* metabolic equivalent.

**Fig. 3:**
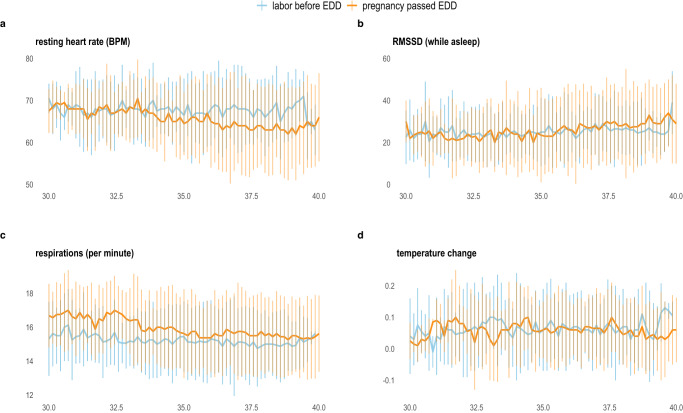
Trends in data derived from smart ring device among 118 pregnant participants from 30 gestational weeks to the estimated delivery date. Data collected from enrollment through birth are included. Median (line) and interquartile range (IQR, vertical range plot) data for (**a**) resting heart rate (beats per minute), (**b**) heart rate variability (milliseconds) using room mean square of successive differences (RMSSD), (**c**) average respiratory rate per minute, (**d**) skin temperature trend (Celcius) from weighted three day rolling temperature average.

We then compared the smart ring derived metrics between the analytic groups: those experiencing labor before the EDD to those whose pregnancies passed the EDD. Figure 3 demonstrates the median and interquartile range values for a subset of the physiological metrics from 30 weeks to 40 weeks of gestation separated by the primary analytic comparison groups. Table 3 shows the differences between these groups’ metrics in mixed effects linear regression models, adjusted for gestational age at the time of analysis. The individuals who had labor induction or pre-labor Cesarean birth prior to the EDD were excluded as the primary research question was focused on the timing of spontaneous labor onset prior to vs. after the EDD and these individuals had a shortened pregnancy due to medical recommendation. After Bonferroni correction for multiple comparisons, only the medium MET metric appeared to differ across the study period (*p* < 0.002). That is, those experiencing labor after the EDD had higher average medium intensity physical activity across the measurement period compared to those who had labor start before the EDD.

**Table 3:** Average smart ring metrics in relationship to pregnancy extending beyond the EDD compared to labor beginning before the EDD.

Metric	Adjusted β	95% CI	*p*-value
MET medium	43.69	17.51–69.86	*0.001*
Medium activity	12.81	21.12–4.50	0.003
Calories active	72.47	17.37–127.56	0.010
Daily movement	1308.4	224.52–2392.27	0.018
Average MET	0.43	0.004–0.08	0.028
Steps	1036.78	−84.40–2157.96	0.07
MET high	7.51	−0.67–15.7	0.072
Caloric expenditure	100.37	−10.95–211.70	0.077
Heart rate average	−2.54	−5.37–0.28	0.078
RMSSD	6.45	−0.89–13.81	0.085
High activity	0.97	−0.15–2.10	0.09
Resting heart rate	−2.18	−4.67–0.66	0.114
Restless sleep	−2.44	−5.50–0.62	0.118
Bed start	−1185.78	−2687.43–315.86	0.122
Sleep efficiency	1.46	−0.40–3.33	0.124
Non wear time	−29.06	−68.13–10.00	0.145
Total sleep	718.07	−271.73–1707.87	0.155
Temperature deviation	−0.008	−0.02–0.005	0.252
Deep sleep	0.144	−0.10–0.39	0.265
Respiratory rate	0.35	−0.29–1.01	0.284
Bed end	−724.82	−2345.75–896.12	0.381
Sleep midpoint	238.21	−346.28–822.71	0.424
MET inactive	0.45	−0.66–1.57	0.426
REM	−0.06	−0.29–0.16	0.59
Rest	4.56	−15.58–24.71	0.657
Low activity	7.18	−25.56–39.92	0.667
MET low	5.13	−18.94–29.22	0.676
Temperature trend	−0.002	−0.02–0.01	0.78
Inactive	3.89	−31.03–38.80	0.827
Sleep onset	−10.93	−111.91–90.04	0.832

Mixed-effects regression model was used for each daily metric, which was regressed onto the groups of participants: those passing their EDD (*n* = 48) versus those with pregnancies ending before the EDD in spontaneous onset of labor (*n* = 39). Adjusted β for gestational age on the day of evaluation. Significance was determined to be *p* < 0.002 (shown as italic).

*RMSSD* root mean square of successive differences, *REM* rapid eye movement, *MET* metabolic equivalent.

We examined smart ring metrics (resting heart rate, temperature change, total sleep, medium activity, RMSSD, average breaths, total caloric expenditure) relative to participant characteristics of pre-pregnancy BMI, fetal sex, maternal age and parity using mixed effects regression, controlling for gestational age day. We did not find any statistically significant findings using a *p* < 0.002.

### Predicting delivery after EDD using physiological metrics

We designed a boosted random forest to predict whether participants in the study would have a longer gestation compared to those who would labor and deliver prior to the EDD. We included all of the features available from the smart ring (Table 2) and we also included the following clinical and demographic features: age, BMI, sex of baby (if known during gestation), and nulliparity (participant’s first birth). In addition, we included the gestational age on the day of the physiological metric as a feature in the model. Participants who delivered via induced labor or pre-labor Cesarean prior to the EDD were excluded. The data set was divided into those participants who gave birth on or before their EDD following spontaneous labor (*n* = 39) and those who had a pregnancy pass the EDD (*n* = 48). The longer pregnancies eventually ended via spontaneous labor (*n* = 31) or after labor induction (*n* = 17).

To train the random forest, we used only data collected prior to 40 weeks of gestation. Specifically, we included gestational days 234–275 (data from 33.4–39.3 weeks of gestation) to do the prediction. We further limited the training dataset by excluding metrics in the 4 days prior to the onset of labor or labor induction. For example, for a person starting labor at 39.0 weeks of gestation (273 days) we included any available data from gestational day 234 to gestational day 269. For those laboring >40 weeks data were included through day 280 only. Our rationale for this approach was to use only data from several days before the due date excluding data whereby labor symptoms in the 4 days just prior to labor were present.

**Fig. 4:**
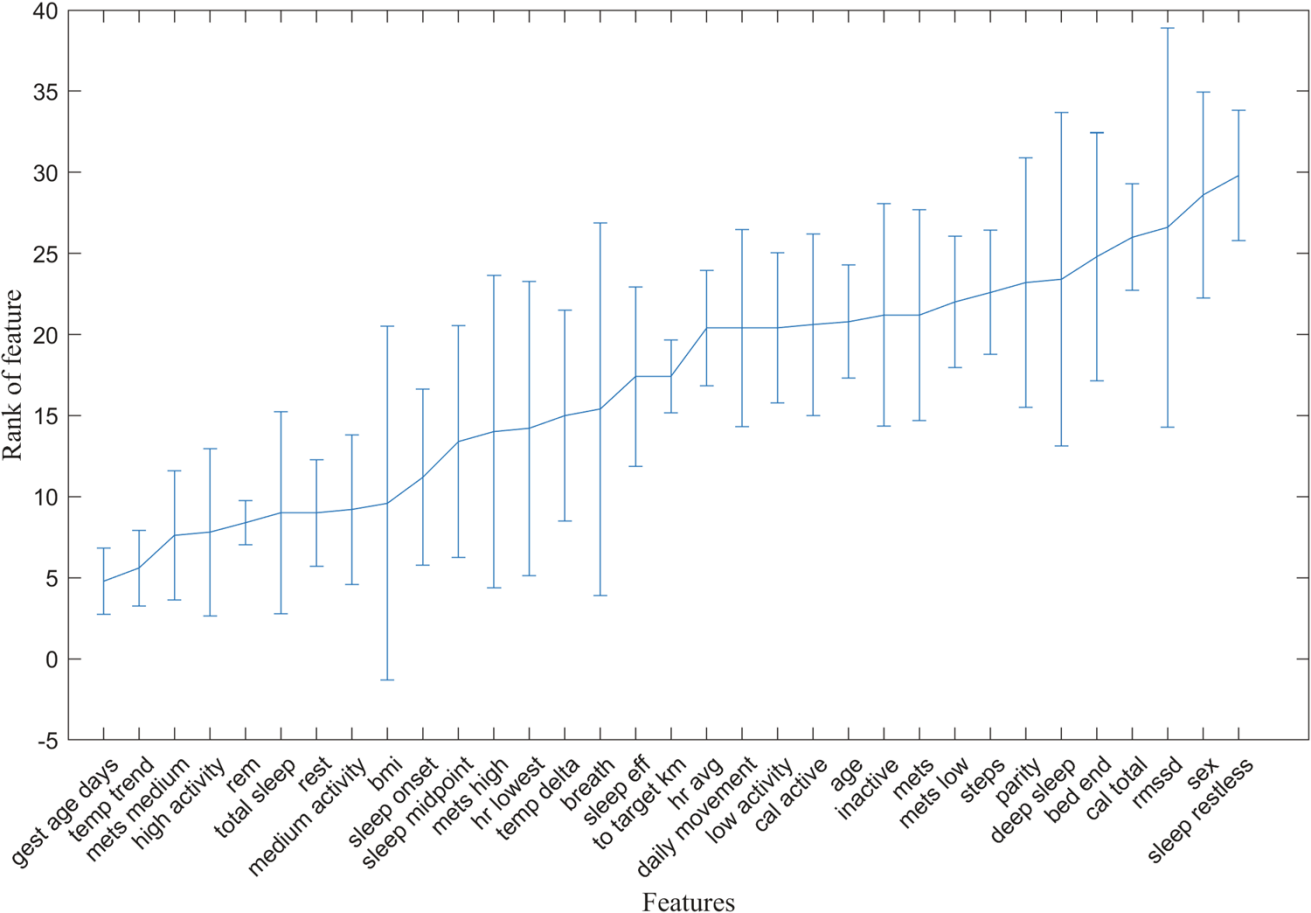
Average predictive rank of features used in boosted random forest model across five runs. Features are shown based on their rank as determined by greedy search in predicting that a pregnancy would pass the EDD. The rank of the feature within the greedy search is shown on the *y*-axis while *x*-axis lists the features from left (best) to worst (right) based on the average of their rank across the five-fold cross validation. The error bars show the standard deviation of each feature’s rank, indicating its consistency at that rank across the five folds.

We performed a greedy search^24^ to determine the combinations of features achieving the highest prediction accuracy as defined by AUC of the ROC curve. To do the greedy search, we started with a single-feature predictor and determined which feature yielded the largest AUC. We then sequentially added features and determined which 2-feature combination yielded the largest AUC. We proceeded with this for 3-feature, 4-feature and up to 33-feature combinations. During the search, we used cross validation to train and evaluate the algorithm whereby we used 80% of the participants for the training and evaluated the accuracy of the predictor on the 20% of the held-out participants. This was repeated 10 times with a different 20% of held-out participants each time. The accuracy for each of the 10 folds was stored. Cross validation of the features was repeated five times. Figure 4 shows how the features ranked across the five runs with the highest ranked features as determined by greedy search are listed from left (best) to right (worst). Each feature’s rank was averaged across the five folds and the standard deviation of the rank is also shown for each feature across the five folds. Supplementary Fig. 1 shows the individual ranking of features across each of the five runs. While gestational age was the highest- ranking feature on average, it was not found to be the highest-ranking features on any of the individual folds (Supplementary Fig. 1). When the random forest was trained only on the gestational age, the performance was significantly worse than when additional physiologic features were included, with a mean sensitivity across 10-fold validation of 0.55 (specificity = 0.56, AUC = 0.58) (Supplementary Fig. 2).

**Fig. 5:**
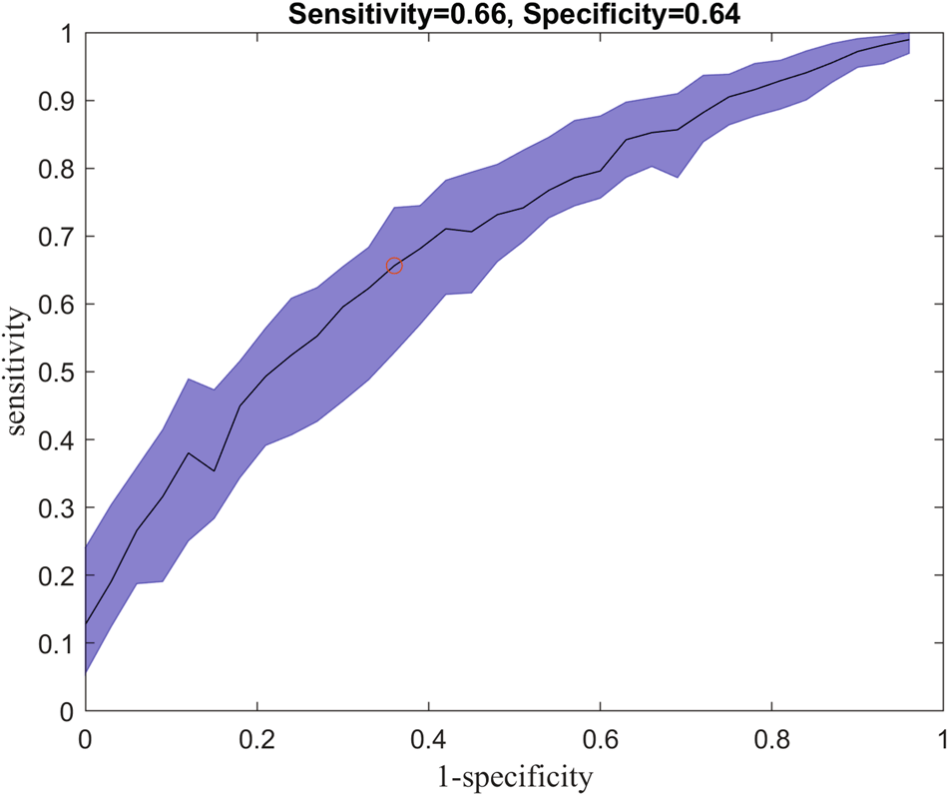
Receiver operating characteristic (ROC) curve showing the sensitivity vs. 1-specificity for predicting if a pregnancy would pass the EDD. The area under the curve on average across the five folds was 0.71 with a sensitivity of 0.66 and specificity of 0.64.

Figure 5 shows the ROC curve for the best feature set of each of the 5 runs using cross-validation data sets. Hyperparameter tuning was done by cross validation and the final number of trees was found to be 100 with a learning rate of 1.0 and a maximum number of splits of 10. We repeated the greedy search 5 times to determine whether the ranked features would change order based on the training/testing sets randomly selected. We found that there was some variability in the ranking of the optimal features, which was most likely due to the fact that many features were highly correlated with each other because they were derived from a small set of sensors on the ring. Regardless, certain features consistently ranked higher than others and yielded a higher prediction accuracy. Overall, the performance of the model was demonstrated by an AUC of 0.71 and optimal sensitivity (0.66) and specificity (0.64) based on the harmonic mean (and IQR) across the 5 runs.

The best ranked features for predicting if a pregnancy will pass the EDD were the gestational age at the time the prediction was made, temperature trend values, MET (medium, high activity) and sleep measures (REM-stage sleep, total, and rest). The next best-ranked features were BMI, sleep onset/midpoint, MET: high, and resting heart rate, temperature change and respiratory rate. Among the least predictive features were sleep efficiency, target kilometers, average heart rate, daily movement, low activity, active calories, participant age, inactivity, average/low metabolic equivalents, total steps, parity, deep sleep duration, end of bedtime, total calories, RMSSD, fetal sex and duration restless sleep.

### Maternal self-reported labor symptoms associated with advancing gestational age but not with mode of labor onset or eventual duration of pregnancy

Participants completed weekly labor symptom surveys administered through automated REDCap^25^ invitations after enrollment. We used mixed effects logistic regression models, with the participant as the random effect, to estimate the likelihood of labor symptoms being reported with advancing gestation. We found that reports of uterine activity, vaginal discharge, menstrual-like cramping and low back pain increased as gestation advanced as a whole (main effects) (Table 4). With two exceptions, interaction models did not demonstrate any significant differences between the group of individuals who experienced spontaneous onset of labor compared to those who did not have spontaneous labor (labor induction or Cesarean birth without labor). Those who eventually had labor begin spontaneously were 25% more likely to report contractions occurring while at rest and 16% more likely to report vaginal discharge compared to those who ended up with labor induction or delivery without labor. Both odds ratios were significant using a *p* < 0.05, which may be spurious findings given the number of comparisons performed in Table 4. No symptom differences were noted between those with pregnancies passing the EDD versus labor starting before 40 weeks. Clinical and demographic data including parity, body mass, ethnicity, age, educational attainment, employment, insurance method, or family income did not differ between those who labored prior to their EDD and those with longer pregnancies (Table 1).

**Table 4:** Symptoms reported by participants during pregnancy in comparison to advancing week of gestation and in relation to labor onset or timing of labor relative to the Estimated Date of Delivery (EDD).

Reported Symptom	Gestational Age	Interaction: Gestational Age × Spontaneous Labor	Interaction: Gestational Age × Pregnancy > EDD
Labor symptoms	OR (95% CI)	OR (95% CI)	OR (95% CI)
Irregular contractions	1.68 (1.53–1.85)***	1.02 (0.86–1.22)	1.03 (0.82–1.29)
Regular contractions	2.11 (1.67–2.67)***	0.87 (0.52–1.45)	0.71 (0.38–1.36)
Contractions at rest	1.72 (1.53–1.92)***	1.25 (1.02–1.55)*	0.98 (0.75–1.29)
Contractions waking at night	1.84 (1.55–2.18)***	1.27 (0.96–1.71)	0.88 (0.61–1.25)
Contractions with movement	1.56 (1.42–1.72)***	1.19 (0.99–1.44)	0.80 (0.60–1.05)
Painful contractions	1.62 (1.40–1.89)***	0.93 (0.69–1.23)	1.15 (0.80–1.63)
Vaginal discharge	1.31 (1.23–1.40)***	1.16 (1.03–1.32)*	0.93 (0.78–1.09)
Low back pain	1.13 (1.07–1.19)***	1.04 (0.94–1.15)	0.96 (0.85–1.08)
Menstrual-like cramping	1.77 (1.61–1.94)***	1.02 (0.86–1.21)	1.12 (0.90–1.40)
Measures of Fatigue/Pain	β (95% CI)	β (95% CI)	β (95% CI)
PHQ-15	0.10 (0.001–0.20)*	−0.01 (−0.22–0.18)	0.14 (−0.08–0.38)
PROMIS^®^- Fatigue SF	0.21 (0.11–0.30)***	0.14 (−0.04–0.33)	−0.19 (−0.41–0.02)
EPDS	0.004 (−0.08–0.08)	−1.87 (−2.73 to −1.00)***^a^	0.35 (−0.42–1.12)^a^
GAD-7	0.12 (0.04–0.20)**	−0.18 (−0.34 to −0.02)*	0.30 (0.13–0.47)**

*PHQ-15* patient health questionnaire 15, *PROMIS®* patient-reported outcomes measurement information system.

*SF* short-form, *EPDS* Edinburgh postnatal depression scale, *GAD* generalized anxiety disorder 7 scale.

*p < 0.05; **p < 0.01; ***p < 0.001.

^a^Not interaction model, no association with advancing gestational age noted.

**Fig. 6:**
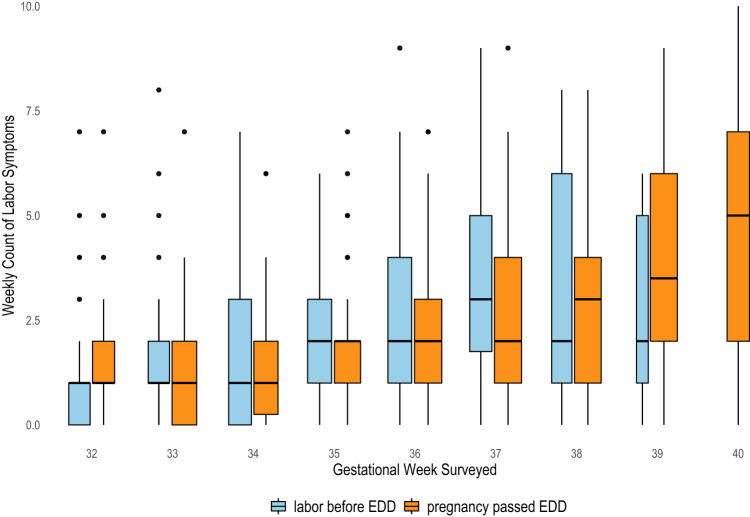
Count of symptoms reported weekly by BioBAYB participants at each week of gestation, comparison between labor beginning before the EDD versus pregnancies passing the EDD. The number of symptoms were summed weekly from a list including contraction patterns (irregular, regular, painful, at rest, waking at night and/or occurring with movement) vaginal discharge, back pain. Box plots center line show mean, bounds of box standard deviation, whiskers interquartile range and dots indicate outliers. Blue boxes are those with spontaneous labor before the EDD and orange represent participants who passed the EDD, laboring spontaneously or with a labor induction. No symptom burden (count) differences were noted between groups.

Finally, no differences were observed between groups in symptom burden (Fig. 6). In sum, these data demonstrate that self-reported symptoms of labor were well-correlated with advancing gestational age, but the likelihood of reporting the symptom was not related to labor occurring spontaneously, nor related to longer vs. shorter gestation.

## Discussion

The purpose of this study was to (1) evaluate how physiological data acquired from a smart ring device during pregnancy are associated with gestational age and (2) evaluate whether these physiological data can be used to predict the natural length of gestation relative to the clinical due date. Our key findings were first that daily physiological metrics (e.g., heart rate, temperature) and sleep and activity measures from the smart ring were statistically related to gestational age, and second that these metrics were useful when used in a random forest model for predicting which participants were likely to pass their EDD compared to those who would give birth earlier in gestation. We also found that increased self-reported labor symptoms correlated to advancing gestational age, but were not helpful predicting which participants were likely to pass their EDD compared to those who would give birth earlier in gestation.

Together, these findings indicate that physiological metrics derived from a smart ring worn during pregnancy are most closely mapped to the advancement of the gestation, particularly given adjustment for multiple comparisons. Comparisons between before/after EDD analytic groups using single metrics were less robust to correction for multiple comparison than the relationship with gestational age, with only medium MET demonstrating significance. In these data, other clinical and demographic characteristics were not robustly associated with the physiological data and participants’ symptoms of labor were also unrelated to timing of labor onset. However, the use of multiple physiological metrics in the boosted random forest model provided for modest prediction of labor beginning before/after the clinical EDD, which was a significant improvement over gestational age alone.

Physiological metrics collected by the smart ring were found to be predictive of timing of birth before or after the EDD. An algorithm that can accurately predict the approximate gestational age of delivery could help to inform clinical decision-making and personal planning around expectations of labor onset. The prediction accuracy of the algorithm may be improved in the future by utilizing data sampled at a higher frequency to capture ultradian patterns^26,27^. A high accuracy algorithm may be useful in planning delivery around term. In cases where balancing uncertainty of risks with ongoing pregnancy are present (e.g., high blood pressure), which may indicate the need for labor induction, knowledge of the approximate time to the natural start of labor would be valuable for providers and patients. Some pregnancies will end naturally weeks before others (Fig. 2)—and knowing the time to labor onset would enable opportunities for personalized recommendations. Forecast of labor timing would also be useful in instances where labor is contraindicated, for example, with placenta previa or certain fetal conditions or if a patient is living a long distance from the hospital.

Our sample was not able to specifically study the prediction of labor starting before term (37 weeks) as we had only one case of spontaneous preterm labor onset at 34 weeks. Future work in larger samples or more high-risk populations are needed to develop models that predict preterm labor. Studies using a WHOOP^TM^ device and HRV data reported there was a prominent rise in HRV around the 33rd week of pregnancy^28^. A preprint using similarly-derived data from WHOOP^TM^ indicated that this inflection was notable around 7 weeks prior to the onset of labor for term and preterm deliveries^29^. Our findings are consistent with this observation from the perspective of increased RMSSD with advancing gestational age. While we did not find RMSSD (heart rate variability) to be a top ranked feature in the boosted random forest model; we did see that RMSSD was related to gestational age in the mixed-effects model. More work is needed on understanding intergroup as well as inter-individual changes across pregnancy and in relation to delivery across the span of gestation.

On average, the most useful physiological metrics in predicting delivery past the EDD included the temperature trend, the metabolic expenditure in the medium range, the high activity metric, and the time spent in REM and total sleep. The most predictive physiological metrics in the random forest varied somewhat based on which validation data sets were used (Figs. 4, 5), which was likely due to the fact that many of the metrics were correlated with each other thus there was not a substantial difference in how these metrics impacted the prediction accuracy. Demographic features including age, fetal sex, and parity ranked poorly in improving model accuracy. The gestational age when a forecast was being done was one of the more predictive variables on average when assessed alongside the smart-ring data, however, adding the physiological metrics increased the model’s predictive ability as a whole. More specifically, the prediction based on gestational age alone was little better than a flip-of-a-coin with and AUC of 0.58. Adding the physiological metrics improved the AUC to 0.71 across the runs.

Further, while temperature trends alone were not significantly related to delivering past the EDD, this metric consistently ranked near the top of features in the random forest. Existing mammalian literature indicate that body temperature changes precede the onset of labor reliably enough to be used as a metric in animal husbandry or to discern the proximity to giving birth in a variety of species (e.g., cow, dog, horse, moose, monkey, orca, rabbit, sheep, squirrel, wolverine)^18–23,30–39^. To our knowledge, this change in temperature as a function of gestational age has not been thoroughly examined in the human literature^40^ and would therefore be useful to study in future research utilizing high temporal resolution. Heart rate variability also has been used to study and predict parturition in pregnant cows^23^.

It is hypothesized that mechanisms governing spontaneous labor in humans could be visible as trends in peripheral physiological output data because physiological metrics (e.g. temperature, heart rate variability) are correlated with changes in inflammation, neuroendocrine or autonomic activity^16^. The transition from pregnancy into labor results from alterations in numerous hormones, gene/protein expression and reproductive tissue modifications^14,15,41,42^. Briefly, placental progesterone production (which maintains uterine quiescence) is progressively subdued by greater placental/fetal corticotropin releasing hormone (CRH) production as well as estriol dominance^43^. The rise of estriol is partly dependent upon the fetus’ nervous system and adrenal gland maturation, which leads to greater dehydroepiandrosterone sulfate (DHEA-S) production^44^. As such, the fetal production of CRH not only contributes to lung maturation through surfactant production, but also impacts the timing of labor onset^45^. Estriol and CRH, as well as inflammatory changes, feed-forward transforming maternal uterine and cervical tissues in preparation for labor^14,46^. Progesterone is a respiratory^47^ and body temperature stimulant^48–50^, and higher progesterone is associated with higher heart rate and lower heart rate variability^28,51–54^. Therefore, the functional progesterone withdrawal^55^ in the lead-up to labor onset could potentially be apparent by tracking peripheral metrics, such as respiration, temperature and heart rate. In addition to these mechanisms, studies with non-human mammalian models also show that the autonomic nervous system (ANS) contributes to labor onset and progress and can therefore be inhibited or stalled by experiences that trigger sympathetic dominance^17,56^. Heart rate variability is a commonly utilized measure of the parasympathetic/sympathetic balance of the ANS^56,57^, which could be monitored non-invasively with wearable sensors^23,28,58^.

Current clinical methods for counseling pregnant individuals on how and when to prepare for labor onset involve considering the current date relative to the EDD, monitoring symptoms, cervical examination (digital or via ultrasound) and general advice based on population-based data from non-specific demographic characteristics (e.g., first pregnancies tend to be a little longer than subsequent pregnancies). Conventional and clinical advice centers on monitoring symptom patterns in anticipation of when labor might be beginning. However, symptoms are typically most useful to diagnose labor in the moment, despite being highly variable from person to person^11,59^. In our study, weekly self-reported symptoms did not vary by timing of labor relative to the EDD or whether labor was spontaneous vs. induced. This finding underscores the difficulty (or flawed assumptions) in using symptoms to help guide prediction of future labor onset in personal or clinical decision making. This observation may be limited by our use of a weekly sampling method. Daily questionnaires may improve observations of subtle changes in labor-related symptoms, though individual burden and attrition may increase as well.

Interestingly, in our sample, few demographic or clinical features were associated with a pregnancy passing the EDD compared to laboring spontaneously before the due date. Our enrollment criteria for the study (generally healthy, low-risk for complications in pregnancy) likely played a role limiting our ability to detect differences in pregnancy complications through the smart ring metrics. In addition, the homogeneity in the sample in terms of educational background and income also likely influenced these null findings. Of the self-reported ethnicity/ancestry, the majority of individuals who identified as Hispanic/Latina were among those giving birth spontaneously prior to the EDD (*p* < 0.05) (*n* = 7 of 10 Hispanic participants), though, overall, the sample was mostly made up of those reporting European ancestry/White identity. Repeating this study in a larger, more diverse sample or among those with higher-risk obstetric histories would be valuable.

Several studies have utilized a variety of clinical^60,61^ or ultrasound derived measures^62–64^ to help predict the future spontaneous onset of labor, while others have used multi-omics data gathered from blood samples across gestation^65^. Clinical use of the fetal fibronectin test is currently used to predict preterm birth (prior to 37 weeks) in high-risk patients, typically presenting with risk factors or labor symptoms. This vaginal swab test used for screening has a sensitivity of 43–92% and specificity of 52–93% in high-risk patients^66^. However, fetal fibronectin has no proven utility in predicting term labor onset. Each of these approaches/tools has a similar objective, to forecast the natural expected length of the pregnancy; however, each method cited above performs with varying degrees of accuracy and requires patients to undergo specific procedures or tests performed at the clinic. A method using maternal remote physiological monitoring would offer the opportunity to evaluate the likelihood of labor starting using non-invasive tools in the person’s home environment. Other approaches for wearable non-invasive labor prediction have included use of electrohysterography (measuring electrical activity from uterine muscle) with or without maternal heart rate data^67,68^, which appear to characterize the early stages of labor itself and may have particular utility in signaling preterm labor when overt symptoms are not perceived.

One of the limitations of the study is that it assessed proprietary metrics acquired from a commercial smart ring. The accuracy of the metrics could not be independently validated by our team; however, there have been prior publications demonstrating the accuracy of its calculation of heart rate variability^69^ and skin temperature^70^. Many of the metrics are derived from a small set of sensors, which means that they are correlated with each other. Another potential limitation for interpreting the sensor data is related to differences in physical activity between groups in which the individuals’ work/home life patterns or exercise routines differ, as these activity habits potentially mediate patterns in other metrics (i.e., heart rate or respiratory rate). Future work can elucidate these differences using experimental or statistical methods. Replication of this approach in another independent physiological dataset gathered in pregnancy is needed to validate these findings. Given our enrollment period was largely limited to the third trimester, future studies should aim for monitoring for a greater proportion of the entire pregnancy. These data will help researchers discern a larger baseline, examine trends across each trimester, and determine the minimum number of days/weeks of data necessary to obtain reliable predictions on the timing for labor onset. We also note the limitations in generalizability, given the sample was largely self-identified as White and a majority had at least a college-level of education. Despite the limitations, the strengths of the investigation include use of a device that was relatively easy/comfortable for the participants to wear; only two participants withdrew from the study because of device discomfort issues. This resulted in high wear time and limited missing data. Our findings are augmented by the use of weekly survey data on labor symptoms to compare to the physiological metric utility. The use of a predictive training/testing methodology allows the data to forecast clinical utility of this kind of physiological data driven approach to labor prediction. Another strength is the use of data gathered prior to the EDD (and several days in advance of labor) which, if replicable or improved upon, could offer a window of time for a person to make important decisions and for their care providers to offer more personalized recommendations or consider alternative testing/treatment if labor was unlikely to begin prior to the EDD.

We demonstrate how multi-modal data derived from a commercially available wearable smart ring device is associated with the maternal physiological state across gestation and how these data can be used to help predict whether birth may occur before or after the clinical Estimated Delivery Date.

## Methods

### Study oversight

The institutional review board for Oregon Health and Science University (Study #20059) reviewed and approved the protocol for this study. This research was carried out in accordance with the Declaration of Helsinki.

### Participants

Participant recruitment strategies included social media advertising across the United States as well as posted paper and digital announcements in the metro Portland, Oregon region. Inclusion criteria included: adults (at least 18 years of age), able to provide written (e-consent model) informed consent^71^ in English who were having a generally healthy pregnancy, at least 26 weeks of gestation (after gestational diabetes testing), had no contraindication for vaginal birth and pre-pregnancy body mass index of less than 40 kg/m^2^. Exclusion for enrollment included: current gestational diabetes, hypertension or uncontrolled thyroid disorders, plans to undergo unlabored Cesarean or to induce labor prior to 41 weeks of gestation, history of ovulatory dysfunction or use of assisted reproductive technology (e.g., in vitro fertilization) for current pregnancy, working rotating or night shifts. A smaller group of participants were enrolled prior to 26 weeks’ gestation if they had a history of prior preterm birth or current twin gestation and the participant otherwise met inclusion criteria.

### Study design and procedures

This study used a prospective observational design. After enrollment, participants completed an online survey including demographic data, health/pregnancy history, social determinants of health and an array of psychometric and symptomatology surveys. Surveys included questions on stress (Perceived Stress Scale), sleep (PROMIS® Sleep Related Impairment—SF), sleep patterns (Munich Chronotype Questionnaire), occupational strains, depression and anxiety scores (Edinburgh Postnatal Depression and Generalized Anxiety Disorder-7), Antenatal Attachment Scale, adverse childhood experiences (ACES), social support (MOS), fatigue (PROMIS®-SF), physical symptoms/pain (PHQ-15), emotional support (PROMIS®-SF) and self-efficacy (PROMIS®-SF).

A weekly survey of labor symptoms was sent to participants and asked the respondent to indicate (Yes/No) if they had experienced any of the following across the last seven days: different patterns of contractions (irregular, regular, contractions while at rest, painful, waking at night with contractions, and/or with movement), low back pain, vaginal discharge or menstrual-like cramping. We also repeated the PROMIS®-SF scales on emotional support, self-efficacy, sleep and fatigue weekly. After baseline, mood and anxiety scores were assessed monthly throughout the study period (GAD-7 and EPDS).

Participants were sent a ring-fitting kit made by the manufacturer (Oura, Finland) which contains eight different ring sizes and were instructed to wear the best-fitting ring for 24 h to ensure comfortable fit and ability to remove it after sleep. Each participant was provided a dummy-coded email address for signing up and syncing their ring with the smartphone application. Data would sync and upload to a cloud-based platform upon opening the Oura App. In the event the app was not opened, the ring would store several days’ worth of data. Rings were charged as needed, typically lasting several days before the application prompted the participant to charge the ring.

Follow-up surveys were sent either weekly or monthly depending on the questionnaire until birth occurred. Upon giving birth, participants notified the study staff via email. A birth experience survey was sent and any clarifying questions were asked by staff via email, when needed. Self-reported outcomes included any pregnancy associated conditions (gestational hypertension, preeclampsia, abnormally low amniotic fluid, or growth restriction etc.). Participants also reported if they experienced labor prior to delivery (versus Cesarean birth before labor began), the date of delivery, mode of birth (vaginal, Cesarean or instrument-assisted vaginal birth), and their newborn(s)’ weight(s). For those who experienced labor, participants reported if the labor began spontaneously versus a labor induction via pharmaceutical (e.g., oxytocin, prostaglandin) or mechanical methods (e.g., artificial rupture of membranes).

### Smart ring data

The Oura Ring is a commercial health tracking device worn on the finger. The Gen2 Oura Ring is equipped with temperature (negative temperature coefficient (NTC)), 3-D accelerometer, and infrared photoplethysmography (PPG) sensors and measures physiological signals, such as heart rate (HR), heart rate variability (HRV), temperature trends, respiration, and movement. The sensors are located in the inner part of the ring on the palm side of the finger and the ring is water resistant up to 100 m. Data is transmitted from the ring to the user’s phone via Bluetooth, and from the phone it is uploaded to the cloud. Users can view their physiological measurements and insights in the Oura App. Participants wore an Oura Ring throughout the study on whichever finger that they could achieve the best fit on the non-dominant hand. The continuous data collection enables the establishment of personalized biometric baselines for each user. Thirty features from the wearable-derived data across gestational age were available from the manufacturer of the smart ring including the following: activity (inactive, rest, low, medium, high, steps, total daily movement), metabolic equivalents (MET) (average, low, medium, high, inactive), calories (active, total), heart rate (average, resting), HRV (root mean square of successive differences (RMSSD)), sleep (start, onset, midpoint, end, efficiency, total, restless, deep, rapid-eye-movement (REM)), average breaths per minute, temperature deviation (weighted average across days) and delta from prior day, and non-wear time.

### Outcomes

Outcomes were assessed across the sample relative to gestational age and then between two groups. The first group was made of participants experiencing the onset of spontaneous labor at or before the EDD, and was compared to the second group, consisting of participants whose pregnancies lasted more than 40 weeks. This dichotomy was chosen as the pregnancies passing the EDD would all have hypothetically started labor eventually if intervention had not been undertaken for another indication. The EDD was reported by the participant and we also recorded the manner in which the EDD was determined (using the last menstrual period or ultrasound). Labor and birth times were self-reported. First, we examine the relationship between gestational age at the time of measurement with each smart ring metric followed by a comparison of the physiological metrics between the groups. Then we report the labor-related symptoms, symptom burden, and pain, fatigue and mood scores in relation to shorter versus longer gestation.

### Statistical analyses

Descriptive baseline data and self-reported birth outcomes were compared between groups having spontaneous labor onset prior to 40 weeks’ gestation versus a longer pregnancy using bivariate statistics (parametric or non-parametric as appropriate).

In assessing whether the physiological metrics varied with gestational age we performed a mixed effects linear regression analysis whereby gestational age in days was the independent variable used to predict each physiological metric when controlling for body mass index (BMI), age of the mother at the start of pregnancy, parity (number of prior births), and sex of the baby if known during pregnancy. Given that some of the metrics were correlated with each other due to the fact that they were derived from a minimal set of sensors, we used a Bonferroni adjustment to divide *p* = 0.05 by the 30 features evaluated with gestational age, to obtain a significance level of *p* < 0.001667 (*p* < 0.002). We also measured differences in the physiological metrics relative to participant characteristics (BMI, parity, maternal age or fetal sex).

Using mixed effects logistic regression, we compared presence/absence of individual symptoms of labor in relationship to advancing gestational age, again using an interaction term for spontaneous labor onset compared to labor that was induced. Next, we tested an interaction term between groups of participants with labor starting before the EDD versus those with longer gestations. Measures of mental health (mood, depression/anxiety), fatigue were compared against gestational age with mixed effects linear regression and interactions. We also examined a weekly symptom burden as the sum of the number of symptoms reported by each participant during each week of gestation and compared the symptom burden at each week from 32–40 weeks between those who experienced induced birth prior to the EDD compared with those who had labor begin spontaneously or those with a longer pregnancy with a Poisson regression model.

### Predictive model

A boosted random forest (Adaboost1) was trained using 10-fold cross validation whereby 80% of the participants were included in the training of the model and for hyperparameter tuning, and 20% were used for testing the accuracy in the test set. The objective was to determine which combination of features yielded the highest cross-validation accuracy on the test set. This was done using a greedy search of optimal features by starting with a single feature and determining the most accurate predictor based on the area under the curve (AUC) of the receiver operative characteristic (ROC) curve. After the first optimal feature was identified, that feature was combined with each additional feature to determine the best combination of two features that would yield the highest AUC. This process was repeated across all of the features.

### Reporting summary

Further information on research design is available in the Nature Research Reporting Summary linked to this article.

### Supplementary information

Supplementary Material

Reporting Summary

## Data Availability

Data gathered in this investigation are subject to data use agreements with parties involved in the study and are therefore not freely available.

## Code availability

Code will be made available upon request to the contact author.

No custom code was generated for these analyses.
