# Supplementary material for: Predicting labor onset relative to the estimated date of delivery using smart ring physiological data
Source: NPJ Digit Med. 2023 Aug 19;6:153. doi: 10.1038/s41746-023-00902-y (PMC10439919; doi:10.1038/s41746-023-00902-y)
Supplement: Supplementary file 1 — Supplementary Material [file 41746_2023_902_MOESM1_ESM.pdf]

Supplementary Material

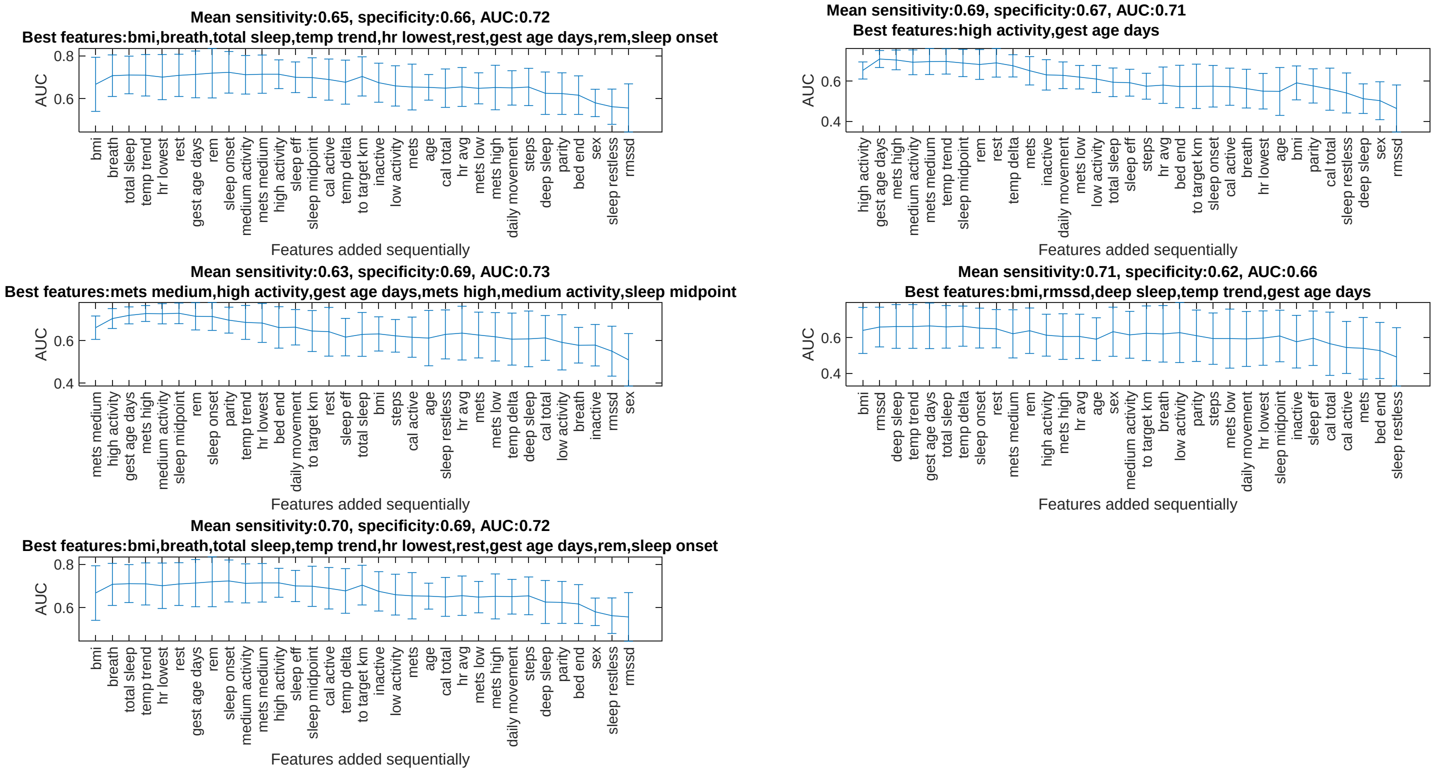

Supplementary Figure 1: Panels 1-5 above show the best ranked features, arranged from left to right, with the left-most feature selected first, in a 10-fold cross-validation on 5 different hold-out sets of the data to determine which features consistently predicted more accurately as determined by higher AUC. The panels show the top ranked features (left to right, best to worst) as determined using greedy search for the five different folds of the data. While certain features tended to consistently be ranked at the top (e.g. temperature trend and MET medium), the best features selected depended a lot on the fold of data. For this reason, we averaged the rank of the features to determine which features appeared to be most relevant consistently across the five folds in their ability to predict if a woman was going to go beyond her expected term. It is not surprising that the features provided incremental benefit over previously selected features, since the features were derived from a very small set of sensors; thus many of the features are highly correlated.

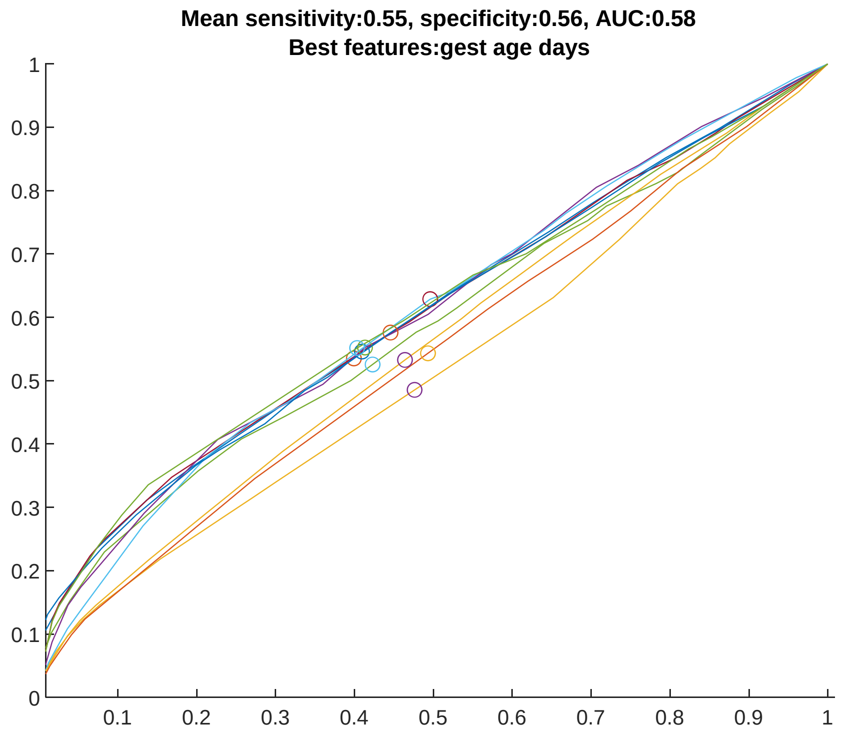

Supplemental Figure 2: ROC curve showing results when gestational age was used as the only feature to predict if a pregnancy would exceed term. The mean sensitivity across 10-fold validation was 0.55, specificity was 0.56 and the AUC was 0.58. While gestational age proved to be a valuable feature in predicting if a pregnancy would go beyond term, it was never the top feature as confirmed in Supplemental Figure 1.
