# Supplementary material for: Predicting labor onset relative to the estimated date of delivery using smart ring physiological data
Source: NPJ Digit Med. 2023 Aug 19;6:153. doi: 10.1038/s41746-023-00902-y (PMC10439919; doi:10.1038/s41746-023-00902-y)
Supplement: Supplementary file 2 — Reporting Summary [file 41746_2023_902_MOESM2_ESM.pdf]

nature portfolio

Corresponding author(s): Elise N. Erickson, PhD, CNM

Last updated by author(s): 04/13/2023

Reporting Summary

Nature Portfolio wishes to improve the reproducibility of the work that we publish. This form provides structure for consistency and transparency in reporting. For further information on Nature Portfolio policies, see our Editorial Policies and the Editorial Policy Checklist.

Statistics

For all statistical analyses, confirm that the following items are present in the figure legend, table legend, main text, or Methods section.

n/a	Confirmed	
☐	☒	The exact sample size (*n*) for each experimental group/condition, given as a discrete number and unit of measurement
☐	☒	A statement on whether measurements were taken from distinct samples or whether the same sample was measured repeatedly
☐	☒	The statistical test(s) used AND whether they are one- or two-sided<br>*Only common tests should be described solely by name; describe more complex techniques in the Methods section.*
☐	☒	A description of all covariates tested
☐	☒	A description of any assumptions or corrections, such as tests of normality and adjustment for multiple comparisons
☐	☒	A full description of the statistical parameters including central tendency (e.g. means) or other basic estimates (e.g. regression coefficient) AND variation (e.g. standard deviation) or associated estimates of uncertainty (e.g. confidence intervals)
☐	☒	For null hypothesis testing, the test statistic (e.g. *F*, *t*, *r*) with confidence intervals, effect sizes, degrees of freedom and *P* value noted<br>*Give P values as exact values whenever suitable.*
☒	☐	For Bayesian analysis, information on the choice of priors and Markov chain Monte Carlo settings
☒	☐	For hierarchical and complex designs, identification of the appropriate level for tests and full reporting of outcomes
☒	☐	Estimates of effect sizes (e.g. Cohen's *d*, Pearson's *r*), indicating how they were calculated

Our web collection on statistics for biologists contains articles on many of the points above.

Software and code

Policy information about availability of computer code

Data collection	REDCap Instruments (Perceived Stress Scale, PROMIS® Sleep Related Impairment—SF, Munich Chronotype Questionnaire, Edinburgh Postnatal Depression, Generalized Anxiety Disorder-7, Antenatal Attachment Scale, ACES, MOS, PROMIS®-SF, PHQ-15); Gen2 Oura Ring App software / ring firmware
Data analysis	Adaboost1 boosted random forest; custom random boosted forest (for comparing longer gestation to those who would labor/deliver prior to EDD)

For manuscripts utilizing custom algorithms or software that are central to the research but not yet described in published literature, software must be made available to editors and reviewers. We strongly encourage code deposition in a community repository (e.g. GitHub). See the Nature Portfolio guidelines for submitting code & software for further information.

Data

Policy information about availability of data

All manuscripts must include a data availability statement. This statement should provide the following information, where applicable:

- Accession codes, unique identifiers, or web links for publicly available datasets
- A description of any restrictions on data availability
- For clinical datasets or third party data, please ensure that the statement adheres to our policy

Data gathered in this investigation are subject to data use agreements with parties involved in the study and are therefore not freely available.

Research involving human participants, their data, or biological material

Policy information about studies with human participants or human data. See also policy information about sex, gender (identity/presentation), and sexual orientation and race, ethnicity and racism.

Reporting on sex and gender	Findings on participants apply only to those of the female sex/ pregnancy research. Sex and gender were self-reported in REDCap surveys, but not relevant to findings and thus not reported as findings. Fetal sex (if known) was determined based on EMR data (N=49 females, N=49 males, N=20 unknown at enrollment), and fetal gender was not considered in study design though was included in the ML analyses.
Reporting on race, ethnicity, or other socially relevant groupings	All socially relevant groupings were self-reported via REDCap surveys. These include: educational attainment, ethnic/ ancestry background, and insurance (employer-based, Affordable Care Act, or state-sponsored). These were used as social determinants of health as they may affect health, outcomes, and risks in relation to pregnancy.
Population characteristics	Age in years (M=32.6, SD=4.1); Gestational age at enrollment (M=30.3, SD=2.9); Nulliparous individuals comprised 57.1% of sample (n=68); pre-pregnancy body mass index (M=24.0 kg/m2, SD=4.1); partnered/married (N=127, all participants); history of preterm birth (n=6, 4.8% of participants)
Recruitment	Social media advertising from around the United States; posted paper announcements in the metro Portland, Oregon region; digital announcements in the metro Portland, Oregon region
Ethics oversight	The institutional review board for Oregon Health and Science University reviewed and approved the protocol for this study.

Note that full information on the approval of the study protocol must also be provided in the manuscript.

Field-specific reporting

Please select the one below that is the best fit for your research. If you are not sure, read the appropriate sections before making your selection.

☒ Life sciences ☐ Behavioural & social sciences ☐ Ecological, evolutionary & environmental sciences

For a reference copy of the document with all sections, see nature.com/documents/nr-reporting-summary-flat.pdf

Life sciences study design

All studies must disclose on these points even when the disclosure is negative.

Sample size	The sample size was not statistically determined a priori given the exploratory nature of the project. We set out to enroll at least 125 participants with the knowledge that attrition due to mode of delivery or induction of labor would occur.
Data exclusions	No data were excluded from the descriptive analyses. The ML model was limited to comparison of spontaneous labor before the EDD and pregnancies extending beyond the EDD due to the primary study question.
Replication	Replication of this experimental or statistical approach in another independent physiological dataset gathered in pregnancy is needed to validate findings related to differences in physical activity between groups in which the individuals; work / home life patterns or exercise routines differ.
Randomization	Randomization was not relevant to this study as there was no experimental intervention (this is a prospective observational study design).
Blinding	Blinding was not relevant to this study as there were no experimental / intervention group allocations.

Reporting for specific materials, systems and methods

We require information from authors about some types of materials, experimental systems and methods used in many studies. Here, indicate whether each material, system or method listed is relevant to your study. If you are not sure if a list item applies to your research, read the appropriate section before selecting a response.

Materials & experimental systems

n/a	Involved in the study	
☒	☐	Antibodies
☒	☐	Eukaryotic cell lines
☒	☐	Palaeontology and archaeology
☒	☐	Animals and other organisms
☒	☐	Clinical data
☒	☐	Dual use research of concern
☒	☐	Plants

Methods

n/a	Involved in the study	
☒	☐	ChIP-seq
☒	☐	Flow cytometry
☒	☐	MRI-based neuroimaging
